# Submicrometer-thick UTe_2_ flake achieved by mechanical exfoliation

**DOI:** 10.1088/1361-6668/ae5742

**Published:** 2026-04-08

**Authors:** Hyeok Yoon, Sungha Baek, Shanta R Saha, Robert E Butera, Johnpierre Paglione

**Affiliations:** 1 Maryland Quantum Materials Center, Department of Physics, University of Maryland, College Park, MD 20742, United States of America; 2 Laboratory for Physical Sciences, 8050 Greenmead Drive, College Park, MD 20740, United States of America; 3 Canadian Institute for Advanced Research, Toronto, Ontario M5G 1Z8, Canada

**Keywords:** mechanical exfoliation, superconductivity, non-van der Waals materials

## Abstract

Incorporating uranium ditelluride (UTe_2_), the leading candidate for topological superconductor, into device platforms is essential for probing its underlying physics and exploring potential applications. The presence of natural cleavage planes in non-van der Waals (vdW) UTe_2_ suggests the feasibility of mechanical exfoliation to obtain flat and thin flakes suitable for device fabrication. In this work, we demonstrate the successful exfoliation of UTe_2_ down to sub-micron thickness and the fabrication of electrical contacts on the resulting flakes. The electrical transport properties measured as a function of temperature and magnetic field are broadly consistent with the expected crystallographic orientations. Furthermore, the exfoliated flakes are sufficiently thin to allow measurements of superconducting critical current and surface oxidation, which are difficult to probe in bulk crystals. These findings elucidate key considerations in UTe_2_ device fabrication and highlight the broader applicability of our exfoliation approach to other non-vdW materials with intrinsic easy-cleavage planes.

Integrating quantum materials into functional devices is essential for unlocking their novel properties that lie beyond the scope of conventional band theory. A central pillar of this effort is to start by producing thin-film materials through bottom-up growth techniques. However, in some cases, achieving optimal thin-film growth conditions that reproduce the properties of bulk single crystals is challenging due to intrinsic chemical complexity, lattice mismatch with substrates, or the presence of defects and disorder. In this case, a top-down approach using miniaturized bulk single crystals provides a practical alternative for small-scale device platform for studying emergent phenomena in mesoscopic, low-dimensional, and interfacial physics.

Several top-down fabrication methods have been developed for producing thin samples from bulk materials. For van der Waals (vdW) materials, exfoliation is the conventional approach, often achieved using simple methods such as the adhesive tape technique to break interlayer coupling [1]. In contrast, for non-vdW materials that have stronger inter-layer coupling, focused ion beam (FIB) techniques have traditionally been used to carve out thin sections from bulk [2]. More recently, advances in exfoliation methods for non-vdW layered materials, such as chemical intercalation, mechanical shearing, and liquid-phase exfoliation, have drawn considerable attention, as they enable the facile and efficient production of thin nanoplatelets by overcoming the stronger interlayer bonding in comparison to vdW materials [3–5].

Uranium ditelluride (UTe_2_) is a material that is well suited for this advanced exfoliation approach, as it possesses easy-cleavage planes along (011) or (0–11) despite not being a vdW material. More importantly, UTe_2_ has emerged as the leading candidate for a topological spin-triplet superconductor, highlighting the need for device-level integration to explore its potential for a fault-tolerant quantum computing device [6–11]. However, the miniaturization of bulk UTe_2_ into small-scale devices is still in its early stages. A key challenge lies in the strong surface degradation of the material when exposed to air or heat [12],which complicates device fabrication. However, considerable efforts have recently focused on miniaturizing bulk UTe_2_. As a top-down approach, it has been demonstrated that a few-micron-thick UTe_2_ flakes can be extracted using FIB techniques, and the resulting flakes preserve key bulk properties such as the superconducting transition temperature and the Kondo coherence temperature [13, 14]. On the other hand, a bottom–up approach to synthesize thin films of superconducting UTe_2_ has not yet been realized, although ongoing research continues to pursue this goal [15]. In this work, we develop a fabrication recipe inspired by the shear-force exfoliation methods to produce submicrometer-thick flakes patterned with electrical leads to characterize its electrical transport properties. We present how we customized conventional exfoliation techniques to accommodate the unique mechanical and chemical properties of UTe_2_. We present the general characteristics of the resulting transport devices and discuss both the potential advantages and limitations of this method.

Figures 1(a) and (b) show, respectively, the custom fabrication process of the UTe_2_ flakes from mechanical exfoliation to electrical contact fabrication and the cross-sectional view of the final device. First, UTe_2_ crystals are mechanically crushed into small fragments and transferred onto a pre-patterned 280 nm SiO_2_/Si substrate. Prior to transfer, the substrate is cleaned via acetone and isopropanol sonication, followed by a 5 min ozone treatment. Conventional exfoliation techniques for vdW materials typically involve heating the sample at elevated temperatures (e.g. 60 °C) to enhance adhesion between the flakes and the substrate. However, this step is omitted in our process, as heating accelerates surface oxidation of UTe_2_ [12]. Instead, we place the flakes onto the substrate, then cover them with weighing paper and apply gentle pressure while sliding. This process naturally promotes the formation of flat flakes via shear forces that mainly cleave along easy-cleavage planes while also improving adhesion between flakes and the substrates.

**Figure 1.**
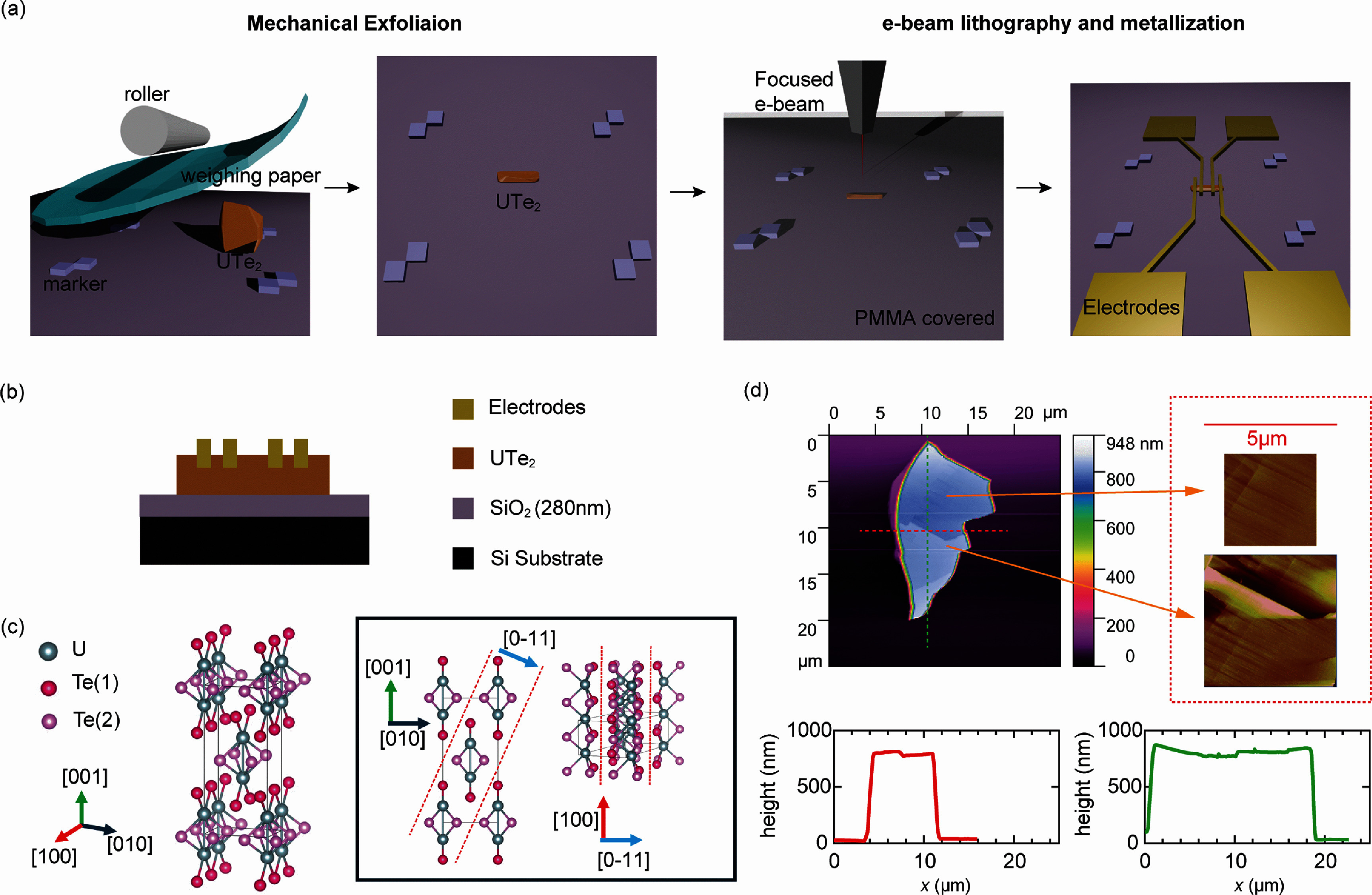
(a) Experimental fabrication process for mechanical exfoliation of UTe_2_ flakes and the formation of electrical contacts via e-beam lithography and metallization. First, a small UTe_2_ lump placed on a 280 nm SiO_2_/Si substrate is pressed using a roller, with clean weighting paper placed between the roller and the UTe_2_ to prevent damage. This process yields small UTe_2_ flakes adhered to the 280 nm SiO_2_/Si substrate, exfoliated along the easy-cleavage planes of UTe_2_. A PMMA layer is then spin-coated, followed by e-beam lithography. Electrodes are deposited after *in situ* Ar ion milling to ensure ohmic contact with the UTe_2_. (b) Cross-sectional view of the device structure. (c) Crystal structure of UTe_2_ showing the easy cleavage planes (011). (d) AFM images of representative flakes obtained, with horizontal and vertical linecuts shown.

The easy-cleavage plane of UTe_2_ lies along the layer in which tellurium atoms form the nearest-neighbor bonds across the plane, as illustrated in figure 1(c). Figure 1(d) describes the best example of flakes that we can obtain using this method. The lateral size of flake can be a few microns to tens of microns, and the thickness of flakes can be a few hundred nanometers (linecuts in the figure 1(d)). We could find thinner flakes below 100 nm based on judgment through color contrast (SI, figure S1) [16, 17]. However, these flakes are generally too small for subsequent lithographic fabrication and are often fully oxidized, leaving no pristine core region.

Going beyond mechanical exfoliation, we proceed to place the electric contacts on the flakes. This step introduces two key considerations that differ from conventional lithography processes. First, we remove the typical PMMA (polymethyl methacrylate) baking step, which is normally performed at 120 °C for 5 min. Skipping the baking step may reduce the lithographic resolution; however, this is acceptable for our feature size of approximately 1 *μ*m. Second, we etch the surface to a sufficient depth using Ar ion milling before depositing the metal contact, using thick PMMA to avoid PMMA hardening. UTe_2_ is highly prone to oxidation under ambient conditions, forming a non-self-limiting oxide layer immediately. Even without a baking process, unwanted oxidation can occur during e-beam lithography steps because of unavoidable exposure to air and chemicals. Based on our experience, achieving acceptable contact resistance requires relatively extensive Ar ion milling, often exceeding 50 nm in depth. However, such aggressive milling can harden the PMMA resist, significantly lowering the yield for metal lift-off. To mitigate this, we found that increasing the PMMA thickness effectively reduces the chances of resist hardening. To achieve thick PMMA layers, rather than applying multiple spin coatings of thinner resist like PMMA A8, we achieve better results using a single layer of thick PMMA A9. This is because, without the baking step, the cumulative thickness of multiple spin coatings does not scale linearly. For instance, a single spin coating of PMMA A8 (A9) yields a thickness of 520 nm (1126 nm), while three consecutive coatings only reach about 712 nm (1998 nm).

Using this customized fabrication process, we fabricated three devices (Device-A, -B, and -C), as presented in figures 2–4. Their typical lateral dimensions range from several micrometers to a few tens of micrometers, with thicknesses on the order of sub-micrometers. Specifically, Device-A (*l* ≈ 30 *μ*m, *w* ≈ 5 *μ*m, *t* ≈ 1 *μ*m), Device-B (*l* ≈ 17 *μ*m, *w* ≈ 5 *μ*m, *t* ≈ 0.5 *μ*m), and Device-C (*l* ≈ 25 *μ*m, *w* ≈ 2 *μ*m, *t* ≈ 1 *μ*m) represent typical examples. Each flake exhibits an elongated geometry, with the long axis likely aligned along the crystallographic *a*-axis, given the wide surface facet is expected to correspond to either the (011) or (0–11) plane. Here, Device-A was measured using a three-point (3-pt) configuration, while four-point (4-pt) resistance measurements were performed for Device-B and C. All devices show a superconducting transition starting around 2 K, as shown in figures 2(c), 3(c) and 4(c). Device-B in figure 3(c), in particular, show a full transition with *I* = 1.5 *μ*A with a plateau at lower temperature. The plateau value is slightly negative rather than zero resistance. We attribute this to a measurement artifact associated with finite common-mode rejection ratio in the voltage amplifier, an effect that becomes more significant as the contact resistance increases.

**Figure 2.**
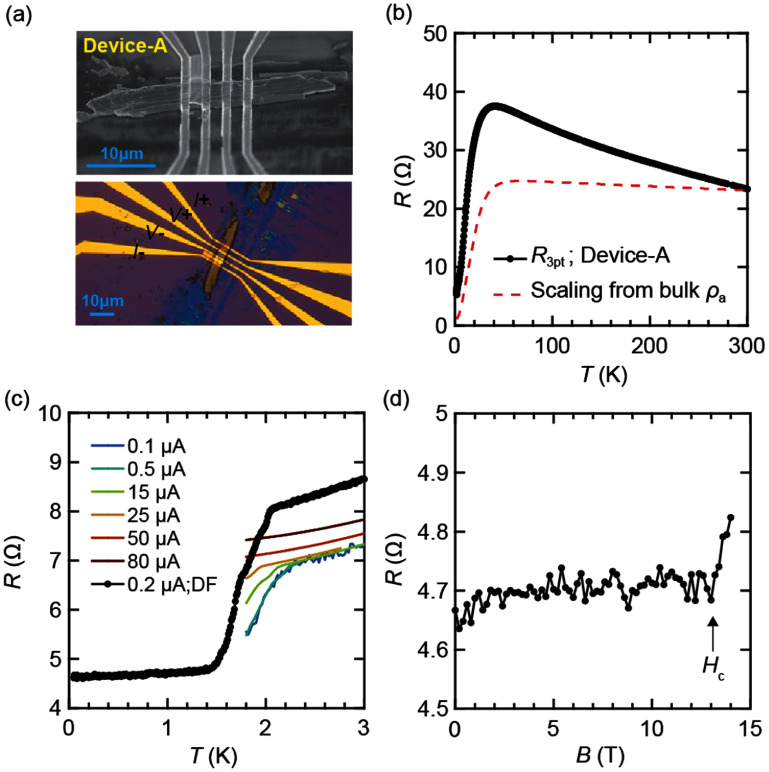
(a) SEM (top) and optical microscope (bottom) images of Device-A. (b) *R*–*T* curve in Device-A from 300 K to 1.8 K (solid black) measured with *I* = 0.5 *μ*A, overlaid with the curve scaled from bulk resistivity along *a*-axis (*ρ*_a_) (dashed red). (c) *R*–*T* curves measured with varying excitation currents down to 1.8 K are shown in different colors. The *R*–*T* curve measured with a fixed current (*I* = 0.2 *μ*A) in a dilution refrigerator down to 50 mK is shown in black, acquired approximately 10 d after the initial measurement. (d) Resistance as a function of perpendicular magnetic field up to 14 T measured at 300 mK.

**Figure 3.**
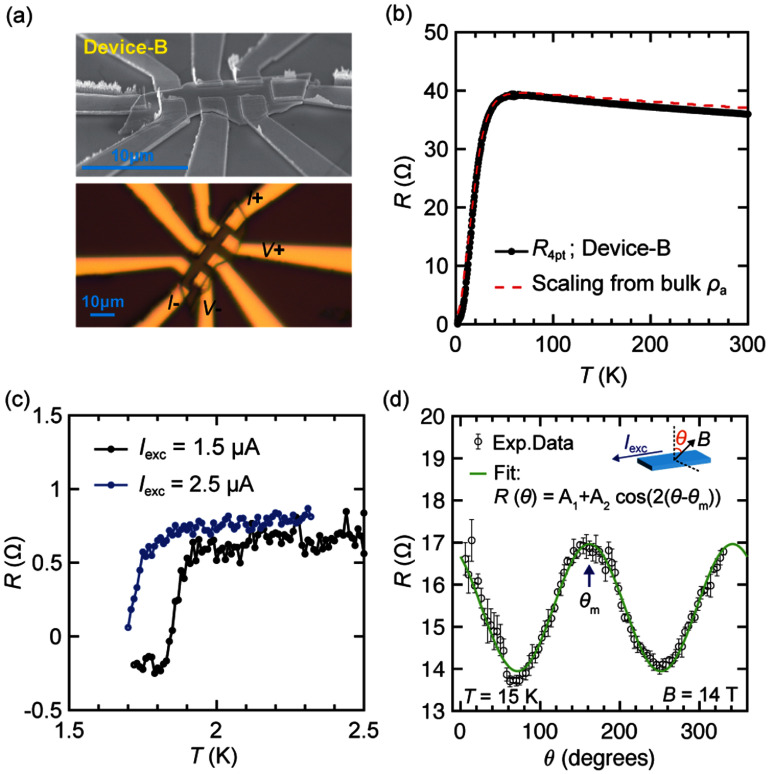
(a) SEM (top) and optical microscope (bottom) images of Device-B. (b) *R*–*T* curve in Device-B from 300 K to 1.8 K (solid black), overlaid with the curve scaled from bulk resistivity along *a*-axis (*ρ*_a_) (dashed red). (c) *R*–*T* curves measured with different excitation currents (1.5 *μ*A and 2.5 *μ*A). (d) Angle dependence magneto-resistance. Current is applied along *a*-axis, and the magnetic field (14 T) is rotated around the *a*-axis, as shown in the inset. *θ* = 0 is when the field is applied perpendicular to the naturally cleaved facet of (011).

**Figure 4.**
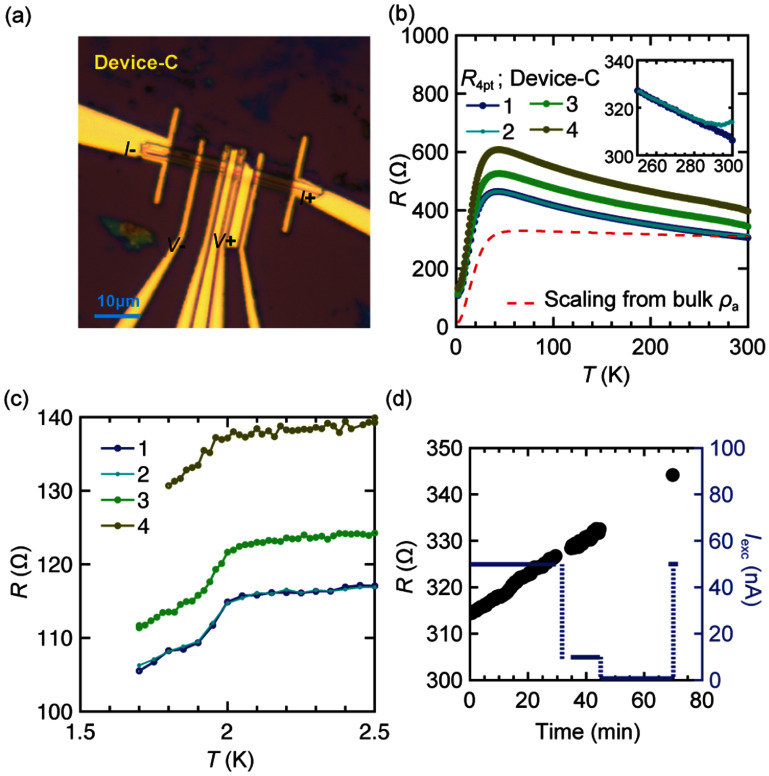
(a) Optical microscope image of Device-C. (b) *R*–*T* curve (Curve-1 to Curve-4), in Device-C from 300 K to 1.8 K overlaid with the curve scaled from bulk resistivity along *a*-axis (*ρ*_a_) (dashed red). Inset focuses on the Curve-1 and 2 between 250 K to 300 K. (c) *R*–*T* curves focusing on the superconducting transition for Curve-1 through Curve-4. The excitation current is fixed at *I*_exc_ = 50 nA. (d) Time evolution of resistance (left axis), with corresponding excitation current variation shown on the right axis. The temperature is fixed at *T* = 300 K.

In addition, the measured resistance values are approximately an order of magnitude larger than the expected bulk resistivity, even in 4-pt configurations. This result suggests that surface oxidation suppresses superconductivity in most of the flake, leaving only about 10% of the volume in a superconducting state. The severe surface degradation is further indicated by the reduction and broadening of the superconducting of superconducting transition in figure 2(c) for Device-A, when remeasured approximately 10 d after the initial measurement. Additionally, in figure 4(c), Device-C exhibits an incomplete transition even at 1.8 K where almost complete transition to zero-resistance is expected in a 4-pt configuration. This behavior is consistent with surface degradation that progressively suppresses superconductivity toward the interior of the flake.

For all devices, the temperature-dependent resistance (*R*–*T*) curves exhibit a characteristic drop near 40 K, indicative of the Kondo coherence temperature, as shown in figures 2(b), 3(b) and 4 (b). For Device-B, the curvature of *R*–*T* curve aligns well with the simulated resistance derived from the bulk resistivity *ρ*_a_. In contrast, Device-A and C exhibit noticeable deviations from the simulated *ρ*_a_ curve, likely due to additional series resistance contributions from the non-superconducting outer oxide layer in the channel. We note that even for Device-B, the exact crystallographic orientation cannot be definitively determined, as *ρ*_b_ also exhibits a temperature dependence similar to *ρ*_a_, albeit with nearly twice the magnitude, which could also fit our data (SI, figure S2(a)). However, a more plausible explanation is that the measured resistivity corresponds to *ρ*_a_, rather than *ρ*_b_ or a linear combination of the two, since the *a*-axis typically aligns with the long axis of cleaved crystals, as is also the case for needle-like single crystals grown by molten-salt flux methods [18]. Despite the ambiguity, the absence of any contribution from *ρ*_c_ is evident, as its inclusion would lead to a markedly different curvature in the *R*–*T* curve due to the peak in *ρ*_c_ around 10 K (SI, figures S2(b) and (c)).

Furthermore, the orientation can be cross-checked by the resistance measurements under a magnetic field. A previous magneto-resistance (MR) study has shown that the angular dependence is most pronounced near the peak in *ρ*_c_ which occurs around 15 K [19]. At this temperature, we find that using bulk samples as a reference with the (011) facet and current along *a*-axis, *ρ*_a_ reaches a maximum (minimum) when the field is aligned along the *b*-axis (*c*-axis) when the magnetic field is rotated in *bc* planes (SI, figure S3). This behavior is also evident in figure 3(d) for Device-B, where the maximum of MR is around when field (14 T) is applied along the expected *b* axis. Here, we fit the angle dependence of MR and deduce the maximum MR occurs at *θ*_m_ = −19° (or 161°) by using the fitting function *R*(*θ*) = *A*_1_ + *A*_2_ cos(2(*θ*− *θ*_m_)). Within the bounds of typical alignment error, the absolute value aligns well with the expected angle between the [011] and [010] directions (23°). In addition, in the case of Device-A, *H*_c2_ ∼ 13 T at 300 mK when the field is applied perpendicularly to the substrate (figure 2(d)), which is close to the expected value along [011] field direction. This confirms that the typical flakes are likely oriented with the *a*-axis as the long axis, and (011) or (0–11) as the cleaved facet, consistent with our expected orientation.

Moreover, as the flake size decreases, the effects of surface oxidation become visibly apparent in the four-probe transport characteristics, as represented in figures 4(b)–(d). Figure 4(b) shows the chronological measurement of *R*–*T* curves (Curve-1 to 4) in the same flake. Curve-1 is measured during cool-down and Curve-2 is measured during subsequent warm-up. Interestingly, as shown in the inset, we clearly see the deviation of these two curves above 280 K. Although the chamber is purged with helium at room temperature when sample is loading and maintains a background pressure of 9 Torr, it is notable that the resistance continues to change. To investigate this behavior in more detail, we measured the resistance as a function of time under varying excitation currents, starting from the room-temperature endpoint of Curve-2, as shown in figure 4(d). The rate of resistance change remains constant regardless of the magnitude of excitation current and continues at the same rate even when the current is completely turned off. This indicates that the resistance change is not due to Joule heating from the excitation current. Therefore, we attribute this ultra-sensitive oxidation behavior to remaining oxygen migration within the flake, which is known to remain mobile down to 200 K [20].

Curve-3, measured during the subsequent cool-down immediately after the time-dependent measurement, displays a significant offset compared to Curve-1 and 2. Following this, the sample was removed from the PPMS chamber, stored in a glove box for 1.5 d, and then reloaded for measurement (Curve-4). Curve-4 also exhibits an apparent offset from Curve-3. In contrast, the contact resistance, characterized by 3-pt configuration, remained unchanged despite the pronounced differences in 4-pt resistance (SI, figure S4). Given that the contacts are embedded tens of nanometers below the surface due to the Ar ion milling, as illustrated in figure 1(b), the variation in resistance from Curve-1 to 4 is attributed to change in resistance of the surface layer which is non-superconducting, rather than the degradation of contact resistance. This interpretation is further supported by figure 4(c), where the superconducting transition shows a similar magnitude of drop across all four curves, despite significant differences in their normal-state resistance values. In addition, the small cross-sectional area of these flakes significantly lowers the critical current, allowing it to be detected at cryogenic temperatures within the measurement limits. As shown in figures 2(c) and 3(c), the critical current near *T*_c_ is suppressed to below several tens of microamperes, a regime that is typically inaccessible in bulk single crystals due to their much larger current capacity. This result highlights the potential of our devices as a platform for investigating mesoscopic phenomena, such as vortex-induced critical current effects [21, 22], to gain deeper insights into the underlying physics of topological and spin-triplet superconductivity. We note that further improvements in fabrication, particularly in removing the surface oxide layer, could enhance the quality and reliability of interpretation of such measurements.

Lastly, on top of demonstrating successful devices, we would like to highlight challenges in current methods that affect device yield, stemming from the intrinsic properties of the materials. As mentioned earlier, using thick PMMA significantly improves device yield during the metal lift-off process. However, despite this improvement, we often observe that the fabricated electrical leads fail to form reliable connections between the top surface of the flakes and the underlying substrate, which can reduce the contact yield. This issue is illustrated in the SEM image of Device-B in figure 3(a), where, among the three electrodes visible on the front side, the middle electrode appears disconnected at the flake’s edge. This finding is consistent with our electrical inspection and likely arises from the substantial thickness of the flakes and the non-uniform or rough bottom surfaces, causing gaps between the flakes and the substrate. Such weak electrical contacts at the flake edges are a major source of vulnerability to electrostatic discharge damage. Another important consideration is preventing oxidation during fabrication, which can be challenging. Since the flakes are thick, capping only the top surface is insufficient. To fully protect the flakes, all sides must be encapsulated, requiring an isotropic deposition of capping materials. Developing and finding such a method would be a crucial next step in advancing UTe_2_ device fabrication.

In summary, we developed a shear-force method to produce submicrometer-thick UTe_2_ flakes, reliably cleaved along easy-cleavage planes and adhered to the 280 nm SiO_2_/Si substrate. By tailoring the contact process to the material’s chemical properties, we successfully fabricated electrical contacts, which is rarely achieved for non-vdW exfoliated flakes. Transport measurements verify the anticipated flake orientation, and the small size of these flakes allows direct investigation of critical current and oxidation effects. We also discuss the advantages and challenges of this method from a materials science perspective. This approach paves the way for advanced UTe_2_ device fabrication via secondary patterning and offers a versatile route for studying other non-vdW crystals with easy-cleavage planes, which holds even greater potential for less air-sensitive materials.

## Data Availability

All data that support the findings of this study are included within the article (and any supplementary files).

## References

[1] Novoselov K S, Geim A K, Morozov S V, Jiang D, Zhang Y, Dubonos S V, Grigorieva I V, Firsov A A (2004). Electric field effect in atomically thin carbon films. Science.

[2] Moll P J W (2018). Focused ion beam microstructuring of quantum matter. Annu. Rev. Condens. Matter Phys..

[3] Jiang K, Ji J, Gong W, Ding L, Li J, Li P, Li B, Geng F (2022). Mechanical cleavage of non-van der Waals structures towards two-dimensional crystals. Nat. Synth..

[4] Balan A P (2022). Non-van der Waals quasi-2D materials; recent advances in synthesis, emergent properties and applications. Mater. Today.

[5] Yang M, Schoop L M (2024). Friends not foes: exfoliation of non-van der Waals materials. Acc. Chem. Res..

[6] Ran S (2019). Nearly ferromagnetic spin-triplet superconductivity. Science.

[7] Ran S (2019). Extreme magnetic field-boosted superconductivity. Nat. Phys..

[8] Jiao L, Howard S, Ran S, Wang Z, Rodriguez J O, Sigrist M, Wang Z, Butch N P, Madhavan V (2020). Chiral superconductivity in heavy-fermion metal UTe_2_. Nature.

[9] Bae S, Kim H, Eo Y S, Ran S, Liu I, Fuhrman W T, Paglione J, Butch N P, Anlage S M (2021). Anomalous normal fluid response in a chiral superconductor UTe_2_. Nat. Commun..

[10] Gu Q (2023). Detection of a pair density wave state in UTe_2_. Nature.

[11] Gu Q (2025). Pair wave function symmetry in UTe_2_ from zero-energy surface state visualization. Science.

[12] Yoon H, Eo Y S, Park J, Horn J A, Dorman R G, Saha S R, Hayes I M, Takeuchi I, Brydon P M R, Paglione J (2024). Probing *p*-wave superconductivity in UTe_2_ via point-contact junctions. npj Quantum Mater..

[13] Helm T (2024). Field-induced compensation of magnetic exchange as the possible origin of reentrant superconductivity in UTe_2_. Nat. Commun..

[14] Zhang L, Guo C, Graf D, Putzke C, Bordelon M M, Bauer E D, Thomas S M, Ronning F, Rosa P F S, Moll P J W (2025). Electronic dimensionality of UTe_2_.

[15] Tereshina-Chitrova E A, Vališka M, Huber F, Gouder T (2023). Synthesis and *in-situ* XPS study of U-Te thin films.

[16] Golla D, Chattrakun K, Watanabe K, Taniguchi T, LeRoy B J, Sandhu A (2013). Optical thickness determination of hexagonal boron nitride flakes. Appl. Phys. Lett..

[17] Ni Z H, Wang H M, Kasim J, Fan H M, Yu T, Wu Y H, Feng Y P, Shen Z X (2007). Graphene thickness determination using reflection and contrast spectroscopy. Nano Lett..

[18] Sakai H, Opletal P, Tokiwa Y, Yamamoto E, Tokunaga Y, Kambe S, Haga Y (2022). Single crystal growth of superconducting UTe_2_ by molten salt flux method. Phys. Rev. Mater..

[19] Eo Y S (2022). c-axis transport in UTe_2_ : evidence of three-dimensional conductivity component. Phys. Rev. B.

[20] Scheiber P, Fidler M, Dulub O, Schmid M, Diebold U, Hou W, Aschauer U, Selloni A (2012). (Sub)surface mobility of oxygen vacancies at the TiO_2_ anatase (101) surface. Phys. Rev. Lett..

[21] Dew-Hughes D (2001). The critical current of superconductors: an historical review. Low Temp. Phys..

[22] Tokiwa Y (2023). Anomalous vortex dynamics in the spin-triplet superconductor UTe_2_. Phys. Rev. B.

